# Sodium into γ-Graphyne Multilayers: An Intercalation Compound for Anodes in Metal-Ion Batteries

**DOI:** 10.1021/acsmaterialslett.4c01119

**Published:** 2024-09-12

**Authors:** Massimiliano Bartolomei, Giacomo Giorgi

**Affiliations:** † Instituto de Física Fundamental, Consejo Superior de Investigaciones Científicas (IFF-CSIC), Serrano 123, 28006 Madrid, Spain; ‡ Department of Civil and Environmental Engineering (DICA), Università degli Studi di Perugia, Via G. Duranti 93, 06125 Perugia, Italy; ¶ CNR-SCITEC, I-06123 Perugia, Italy; § CIRIAF - Interuniversity Research Centre, University of Perugia, 06125 Perugia, Italy

## Abstract

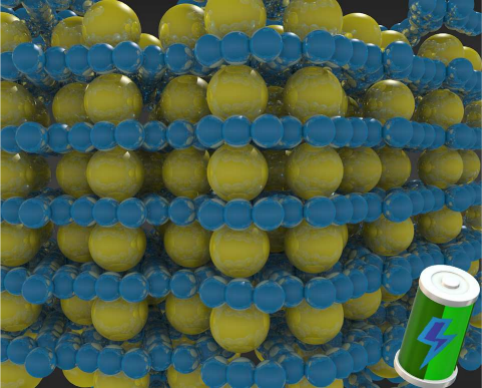

The bulk synthesis of γ-graphyne has been recently achieved and evidenced a multilayered structure, which suggests its potential exploitation as a substitute of graphite-based anode materials for metals heavier than lithium (Li). In fact, each of its regular pores of sub-nanometric size features an optimal environment for hosting a single sodium (Na) ion, as reported here by means of accurate electronic structure calculations. We show that the graphyne*/*Na ion coupling mimics that found on the graphene*/*Li ion in terms of metal-single layer interaction and equilibrium distance. More importantly, in contrast to what is found for graphite, we demonstrate that graphyne intercalation compounds with Na are thermodynamically stable and feature an optimal storage capacity of 372 mAh·g^–1^. These findings, together with a limited crystal structure expansion upon Na intercalation, a low metal diffusion barrier as well as high electrical conductivity, pave the way to the development of novel graphyne-based anodes for efficient Na-ion batteries.

Nowadays lithium-ion batteries (LIBs) represent a well-established source of energy, and since their introduction in the market in 1991^1^ their role has become increasingly critical in several areas of current society, covering from myriad portable electronic devices to electric vehicles.^2−4^ A commercial rechargeable LIB uses a graphite host for Li-ions intercalation as the anode material^5^ owing to its stability, low cost, high conductivity, and good cycle life, which allows reaching a maximum (theoretical) ion storage capacity of 372 mAh·g^–1^, corresponding to a stoichiometry of LiC_6_ for the anode compound. However, considering the increasing need for large-scale applications, finding a substitute for lithium to meet the growing demands for rechargeable batteries is paramount because of the high cost and limited (and unevenly distributed) sources of the lightest alkali element. Moreover, an alternative to Li is also desirable due to the current challenges to be faced with LIB, which span from overheating concerns and low diffusion rates to dendrites growth and capacity fading. Therefore, other metal-ion batteries have been explored^6^ in graphite-based anodes, and both earth-abundant sodium and potassium are, in principle, good alternatives, since they are readily available at a very low cost. Indeed, it was only recently^7,8^ that scientists demonstrated a highly reversible storage of Na and K ions through graphite intercalation but associated with less favorable stoichiometry compounds such as NaC_20_ and KC_8_ as well as with fast capacity fading, probably due to large volume changes over cycling. The rather poor storage capacity of Na in graphite with respect to that of K is, in principle, surprising, since one would expect that alkali ions intercalation would expand the graphite interlayer spacing of an amount directly depending on the metal ionic radius, leading to a higher strain energy for graphite together with a less favorable formation energy for the heavier species. Indeed, this is not the case,^9^ and it so far limits the use of graphite as anode for Na-ion batteries. The origin of this special behavior for Na has been a matter of debate in the past decade,^10^ and it has been recently revisited and finally theoretically assessed:^11,12^ the explanation arises from the competition between two different contributions, i.e., the alkali atom ionization energy and ion-substrate coupling, which have opposite trends along the column of the periodic table. In fact, while the ion-substrate adsorption is more favorable for Na with respect to K, the opposite is found for the ionization energy which in turn benefits to a larger extent the heavier alkali, globally leading to a more negative formation energy for the latter.

This analysis suggests that, in order to improve the Na-ion storage in new materials designed to replace current anodes, the interaction of the metal with the substrate should somehow be enhanced with respect to that of pristine graphite. Also, it is desirable that such materials should maintain a graphite-like layered structure in order to avoid drastic volume change upon insertion^13^ and to allow a facile diffusion in between the two-dimensional (2D) layers. Actually, expanded graphite^14^ has also been recently experimentally proposed as an anode material for Na-ion batteries (NIBs) since the larger interlayer distances obtained by means of a two-step oxidation-reduction process provide more favorable conditions for Na-ions intercalations and allow obtainment of a maximum reversible capacity of 284 mAh·g^–1^.

In this paper we follow the idea of using porous derivatives of graphene such as γ-graphynes^15^ as substitutes for graphite-based anodes, capable of efficiently hosting Na-ions. Indeed, γ-graphynes^15^ represent, in principle, ideal platforms for the design of NIB anodes: in fact, their intrinsic regular porosity, determined by the constituting sp- and sp^2^-hybridized carbon atoms, could allow an easier metal placement closer to the substrate plane and their 2D structure is also amenable for stacking through π–π interactions in a 3D graphite-like layered arrangement. The story of the γ-graphynes family dates back to the late eighties, when they were first theoretically predicted,^16^ but it was just in 2010 when the synthesis of graphdiyne was reported.^17^ Graphdiyne, as the other members of γ-graphynes, is characterized by regular triangular pores of sub-nanometric size, as a consequence of diacetylenic chain linkages connecting repeating patterns of carbon hexagons. Recently, also graphtetrayne (featuring tetra-acetylenic chains) was synthesized,^18^ and in the last years also the experimental assembly of graphyne (featuring monoacetylenic chains) was also reported by means of mechanochemical,^19^ dynamic covalent chemistry polymerization^20^ and scalable cross-coupling polymerization^21^ processes. Since their first report there has been a huge increase of experimental and theoretical studies on γ-graphynes, where several applications have been proposed in gas separation^22−29^ as well as catalysis,^30^ electronics, batteries, solar cells, etc.^28,31−33^ As for the application for NIBs batteries, it is worth noting that, as a result of computations^34^ at the density functional level of theory which suggested a very promising maximum storage capacity of 744 mAh·g^–1^ for Na intercalated in graphdiyne multilayers, the use of bulk graphdiyne powder as an anode material has been recently attempted.^35^ However, the resulting experimental maximum reversible storage capacity of 261 mAh·g^–1^ is far from the ideal estimated value, probably due to the powder nature of the sample featuring microporous and mesoporous structures. Therefore, further investigations on the use of γ-graphynes as anode materials are desirable, and we focus here on graphyne.^36^ Indeed, the very recent bulk synthesis^20,21^ of the latter could pave the way to its large scale production and manipulation and evidenced a crystalline multilayered structure with an interlayer distance of 0.35 nm, which is slightly larger than that of graphite (0.335 nm) and should favor both intercalation and diffusion of alkali ions heavier than Li, such as Na.

As stressed above, one of the key points determining the unfavorable storage of Na ions in graphite is the ion coupling with the substrate, and to better assess and understand such a contribution, we focus first on a finite 2D model of the carbon host. Accordingly, we have performed benchmark *ab initio* calculations at the second-order Møller–Plesset perturbation theory (MP2) level of theory (together with large basis sets) to obtain an accurate estimation of the interaction of both Li^+^ and Na^+^ ions with coronene (C_24_H_12_), which can be considered the smallest (as well as the most computationally affordable) approximation of a graphene sheet. We have previously demonstrated^37^ that the MP2 level of theory is as capable of accurately reproducing the cation−π interaction as that predicted by CCSD(T) calculations for the K^+^-benzene dimer, and here we have validated the same approach also for the Li^+^-benzene and Na^+^-benzene systems, as shown in Figure S1 of the S.I. Thus, in Figure 1 we have reported the MP2 interaction energy profiles of the alkali ions perpendicularly approaching the geometric center of coronene, corresponding to the “hollow” configuration, which is more favorable for the ion adsorption and intercalation. As expected, the binding energy with the smaller Li^+^ ion is clearly enhanced by ∼40% with respect to that for Na^+^ and by more of 70% if compared to K^+^ (see Figure 2 of ref (37)). More importantly, we can also observe that the equilibrium distance for the interaction with Li^+^ ion slightly overestimates the graphite half interlayer distance (shown as a vertical dashed blue line), which marks the optimal region for ion intercalation. That is not the case for Na^+^, which indeed places itself preferably at about 2.4 Å from the carbon plane, while in the proximity of the graphite half interlayer distance the corresponding energy profile becomes quickly repulsive.

**Figure 1:**
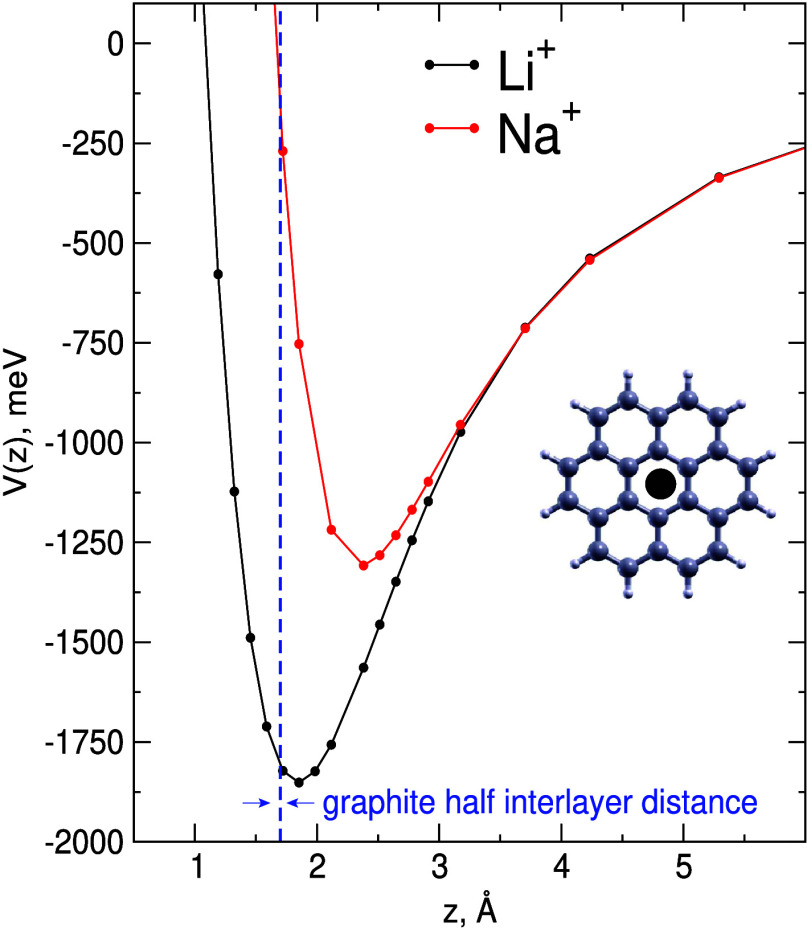
Benchmark interaction energies obtained at the MP2 level of theory for Li^+^ and Na^+^ ions perpendicularly approaching the geometric center (“hollow” configuration) of coronene (see the inset). z represents the coordinate perpendicular to the molecular plane while the blue dashed vertical line corresponds to the graphite half interlayer distance (0.168 nm) and marks the optimal region for ion intercalation.

Such results allow us to rationalize why Na^+^ ions hardly place themselves in between graphene layers: in fact, if compared with Li^+^, the interaction with the carbon plane is reduced by ∼40% and at the same time their intercalation would require a larger deformation of graphite due to a noticeable increase of the interlayer spacing. These findings possibly indicate also the route to validate those layered materials capable of better hosting heavier metal ions through their intercalation. It is at this point that we consider the recently synthesized graphyne and the interaction energies between its molecular precursor and the Na^+^ ion, as presented in Figure 2 together with those for the coronene-Li^+^ ion couple. Hexadehydrotribenzo[12]annulene is considered the smallest molecular precursor of graphyne which indeed correctly reproduces the size and features of the related triangular nanopore (see upper part of right panel of Figure 2)).

**Figure 2:**
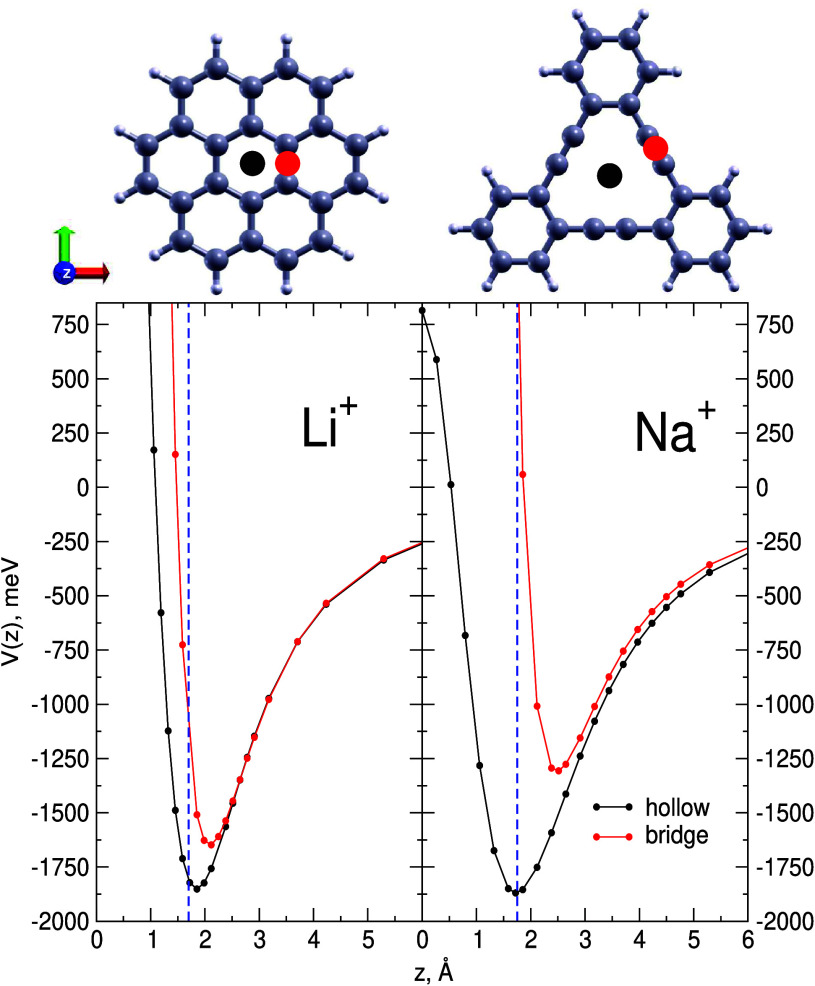
Left panel: benchmark MP2 interaction energies for the Li^+^ ion perpendicularly approaching the inner ring of coronene in the “hollow” and “bridge” configurations (see the upper part). The blue dashed vertical line corresponds to the graphite half interlayer distance (0.168 nm). Right panel: as in left panel but for the Na^+^ ion perpendicularly approaching the graphyne pore here represented by the hexadehydrotribenzo[12]annulene (see upper part). In this case, the “hollow” configuration is that perpendicularly pointing toward the geometric center of the pore while the “bridge” one is that pointing toward the midpoint of the pore side (see upper part). The blue dashed vertical line here corresponds to the bulk graphyne half interlayer distance (0.175 nm).

Moreover, the comparison with the corresponding interaction energies obtained for the adsorption on coronene is particularly significant since both planar molecular prototypes are composed of the same number (24) of carbon atoms. One can notice that for the “hollow” configuration, which in the case of graphyne corresponds to the perpendicular approach to the pore geometric center, both the binding energy and equilibrium distance for the graphyne pore-Na^+^ coupling (right panel) are mimicking those for coronene-Li^+^ (left panel). Moreover, the graphyne pore-Na^+^ equilibrium distance now matches the vertical dashed blue line, which corresponds to the experimental half interlayer distance^20,21^ for bulk graphyne and which again suggests the optimal region for ion intercalation. As for the energy profiles for the “bridge” configuration, as expected, their equilibrium distance is shifted at larger z values, and that is more evident for the graphyne pore–Na^+^ coupling (due to the larger ionic radius of the heavier alkali and larger size of the C–C triple bond), where a shallower well can also be appreciated. Indeed, the energy gap between the “hollow” and “bridge” profiles around their minimum is also of interest since it can provide a rough estimation of the diffusion barrier for ion displacements parallel to the 2D carbon plane: such a barrier is estimated to be around 0.24 and 0.55 eV for Li^+^ over graphene and Na^+^ over graphyne, respectively. Moreover, it can also be deduced that Na^+^ ion diffusion through the graphyne pores is quite unlikely since the corresponding barrier, estimated as the energy difference between the interaction potential at the minimum and that for *z* = 0 of the “hollow” profile reported in the right panel of Figure 2, assumes a value larger than 2.5 eV. Therefore, the results reported in Figure 2 suggest that the intercalation of Na^+^ ions in bulk graphyne could be energetically feasible since there is a noticeable enhancement of the interaction for the ion–substrate coupling together with a related equilibrium distance matching the optimal interlayer spacing region, that is that found in the pristine multilayer carbon material.^20,21^ Moreover, it is also suggested that the ion diffusion would occur exclusively along the 2D plane, although it is in principle more impeded with respect to that for Li^+^ in graphite.

However, to better assess such predictions, a proper validation is needed by means of a periodic model, which is more suited to describe the bulk material and its 3D properties. To do that, a check of a suitable density functional to be used in periodic calculations has been first performed as shown in Figure S2 of the S.I. where we have added to the results in Figure 2 those obtained at the PBE-D3(BJ) level of theory: it can be noticed that both equilibrium distances and well depths are well reproduced by the density functional theory (DFT) estimations although with a slight interaction overestimation in the minimum region (see S.I. for more details).

In order to assess the thermodynamic stability of the binary graphite intercalation compound (GIC) and graphyne intercalation compound (GYIC), which are composed of the layered carbon host and the intercalated alkali ion guest, we can rely on the calculation of the formation energy, which refers to the process sketched in Figure 3 and defined from previous literature as^38^
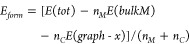
1where *E*(*tot*) is the total energy of the binary GIC and GYIC in their stage-I crystal structure, that is with any contiguous intercalated metal layers separated by one carbon layer; *E*(*bulkM*) is the energy per M atom for the bulk metal (Im 3̅m, s.g. 229, *Z* = 2);^39^*E*(*graph-x*) is the energy of C in pristine graphite or multilayer graphyne (according to the case, both in the AB stacking), and *n*_*M*_ and *n*_*C*_ are the number of M and C atoms in the compound. In calculating *E*_*form*_ for any M-GIC and M-GYIC system, we first keep constant the stoichiometric formula M_*n*_C_*m*_ according to the *n*:*m* ratio equal to 1:6, which corresponds to the maximum theoretical storage capacity found for lithium (LiC_6_)^38,40−42^ in Li-GICs. This allows us to perform a consistent comparison of the intercalation process of the alkali metal in both graphite and γ-graphyne multilayers. As in the case of graphite,^43^ the intercalation of the alkali metal atoms induces in the graphyne multilayer a transition from the AB to AA stacking of the adjacent carbon layers, as assessed from the stability of the GYIC in different configurations (see S.I. for more details).

**Figure 3:**
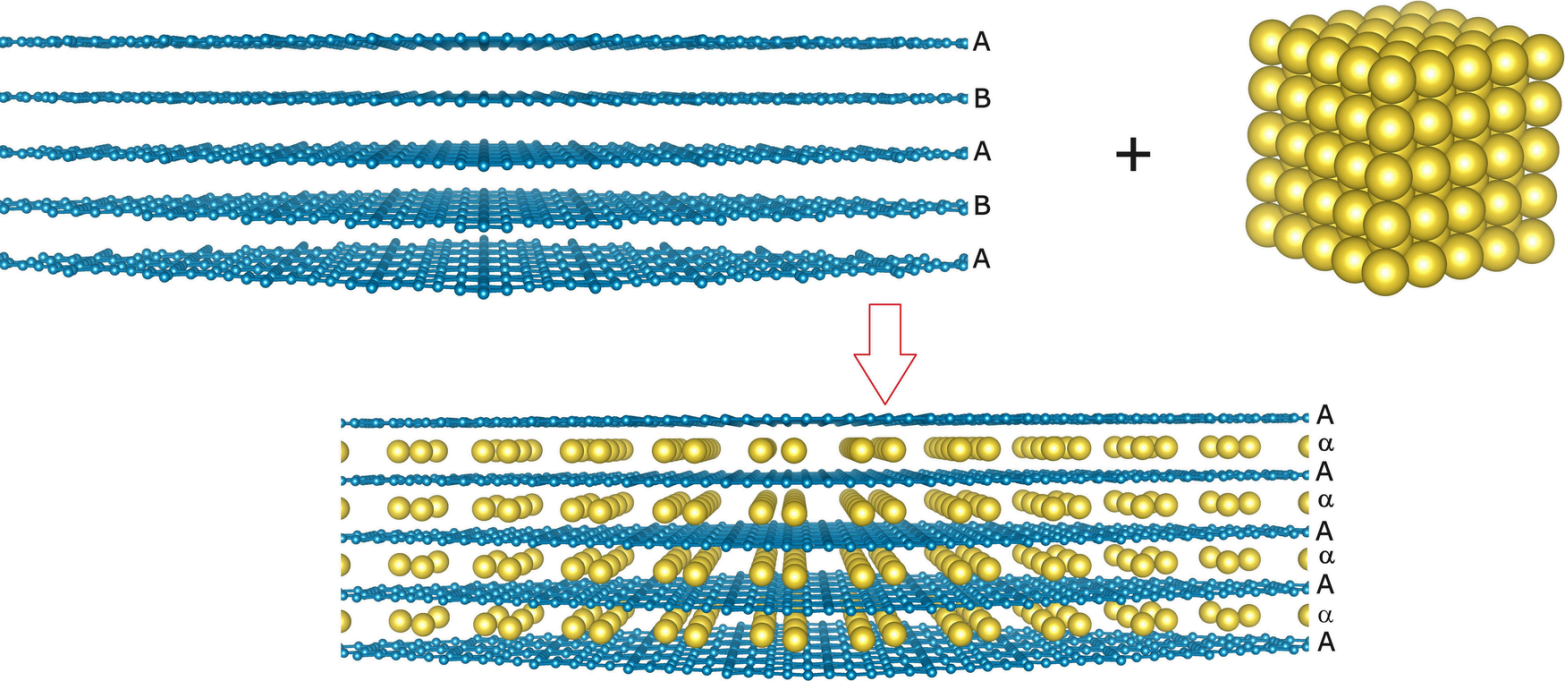
Formation process of Na-GYIC [gold, Na atoms; light blue, C atoms]. The intercalation process here leads to the stage-I binary GYICs with the intercalated metal layers separated by just one carbon plane together with a transition from the AB to the AαAα stacking of the carbon layers. The same mechanism is extended to alkali-GIC systems when considering graphite.

Equation 1 permits an accurate estimation of the related Gibbs free energy of formation, since for such a process both entropic and pressure–volume contributions are expected^12^ to be negligible for these compounds. Therefore, by exploiting periodic DFT calculations at the PBE-D3(BJ) level, the formation energies for Li-GIC, Na-GIC and Na-GYIC are obtained and reported in Table 1. In particular, the Li-GIC and Na-GIC formation energies are −0.011 and 0.048 eV, respectively, in good agreement with the results reported in ref (38) and therefore confirming the instability of the sodium-graphite intercalation compound. However, in the case of Na-GYIC an exothermic value is found, which even exceeds that for Li-GIC, suggesting therefore that sodium intercalation in multilayered graphyne should be possible. The stability of the Na-GYIC with respect to Na-GIC can be rationalized by invoking the enhancement of the binding energy between the metal and the hosting multilayered substrate. As assessed in previous studies,^12,38^ such a binding energy can be approximated by the sum of three contributions: 1) the ionization of the metal by transferring its charge to the substrate; 2) the interaction between the metal cation and the substrate; 3) the dispersion interaction between the contiguous carbon layers. If we assume for Na the first contribution to be the same, independent from the carbon substrate, most of the gained stability of the Na-GYIC is determined by the increased cation–substrate coupling (see Figures 1 and 2), which is a consequence of the fact that the sodium ion can lie closer to the carbon plane when adsorbed upon the graphyne pore. This has also been confirmed by periodic calculations (see Figure S3 in the S.I.) for which a comparable Na–carbon substrate binding energy is found. It can be expected that such interaction is mostly determined by the electrostatic contribution, which considers that the adsorbed Na atom assumes an ionic character, as demonstrated by the associated Bader^44−47^ charge, which is estimated to be 0.864*e* and 0.873*e* (see Table S1 in the S.I.) for Na-GIC and Na-GYIC, respectively. The Coulomb attraction can be estimated by −14.38/(2*d*) eV, with *d* being the distance between the cation and the substrate,^38^ and it allows prediction of an enhancement of the electrostatic interaction of about 40% due to the reduction of *d* when Na interacts with the graphyne substrate. A favorable but minor contribution is also expected for the dispersion attraction acting between adjacent carbon planes, which should increase due to the reduction of the interlayer distance observed for the Na-GYIC with respect to Na-GIC. The calculated interlayer distances for the different pristine and binary intercalation compounds are reported in Table 1, and it can be appreciated that, in fact, for Na-GYIC it is reduced by about 0.6 Å, with respect to its counterpart for the Na intercalation in graphite. Moreover, we can observe for the pristine multilayer graphyne a predicted interlayer spacing of 3.34 Å, which is similar to that estimated for graphite. More importantly, such a spacing increases by about 10% after metal intercalation, mimicking therefore the expansion predicted and also experimentally observed for Li-GIC. Furthermore, the evaluation of the intralayer C–C bonds evidenced that their elongation is increased of about 1% (see Table S1 in the S.I.) after metal intercalation in Na-GYIC, in analogy with that experimentally found^48^ and estimated for Li-GIC. We believe that these represent very relevant features since a limited deformation of the crystal structure upon metal intercalation is necessary for its possible exploitation as anode to mitigate both thermal and volume effects during the charge/discharge cycles. The dependence of the formation energy on the increasing density of the stored sodium has also been investigated (see S.I. and Figure S5 in there) and evidenced that, from the energetic and structural points of view, the optimal Na-GYIC is indeed featuring a NaC_6_ stoichiometry, which corresponds to a gravimetric storage capacity of 372 mAh·g^–1^.

**Table 1:** Formation Energy (*E*_*form*_) as Calculated from Eq 1 for Binary Graphite Intercalation Compounds (GICs) and Graphyne Intercalation Compound (GYIC) and Interlayer Distance for the Pristine (AB Stacking) Multilayers and after Metal Intercalation (AαAα Stacking)^a^

			interlayer distance (Å)	
binary intercalation compound	stoichiometry	*E*_*form*_ (eV)	pristine	metal intercalation
Li-GIC	LiC_6_	–0.011	3.33 (3.35^49,50^)	3.59 (3.70)^48,51^
Na-GIC	NaC_6_	0.048	(same as Li-GIC)	4.34 (4.60)^52^
Na-GYIC	NaC_6_	–0.160	3.34 (3.48)^21^	3.72

a In parentheses are reported the available experimental values.

Once the thermodynamic stability of the Na-GYIC compound is demonstrated, it is important to assess the mobility of the Na atoms inside the crystal structure in comparison with that of Li counterparts in the reference Li-GIC. First, we point out that, in analogy with Li-GIC, the Na mobility is only possible in the direction parallel to the carbon plane since the barrier for the permeation through the pore is too high (more than 2.5 eV), as shown in the right panel of Figure 2 and Figure S3 of the S.I.

To investigate the metal mobility in Na-GYIC, we have considered a larger prototype of the periodic bilayer consisting of eight pores for each layer (see Figure 4) which are all but one occupied by Na atoms, that is, showing one vacancy on each layer. The diffusion barrier is calculated as the activation energy (*E*_*a*_) of the process illustrated in Figure 4, which depicts the migration of one Na atom located on the top layer toward the adjacent and unoccupied pore. Geometric considerations of the pore structure suggest that the minimum energy path for such a migration necessarily involves the passage on top of the midbond of the acetylenic linkage connecting the involved pores since alternative routes (i.e., passing on top of the benzenic ring) would require a quite larger activation. The calculated barrier amounts to 0.67 eV, which is larger but not far from the estimation of 0.51 eV corresponding to the reference Li-GIC system and obtained by considering an analogous periodic structure, reported in Figure S6 of the S.I. and also showing the related diffusion process. The latter value of the diffusion barrier is indeed comparable with the previous theoretical estimations for Li-GIC (see Table 1 of ref (5)). Therefore, the present predictions are expected to be reliable and the obtained alkali diffusion barrier obtained for Na-GYIC suggests that the metal mobility within the multilayer should be not far from that^53^ for Li-GIC, which in turn has been experimentally estimated to be lower than that^54^ for sodium stored in hard carbon based anodes.

**Figure 4:**
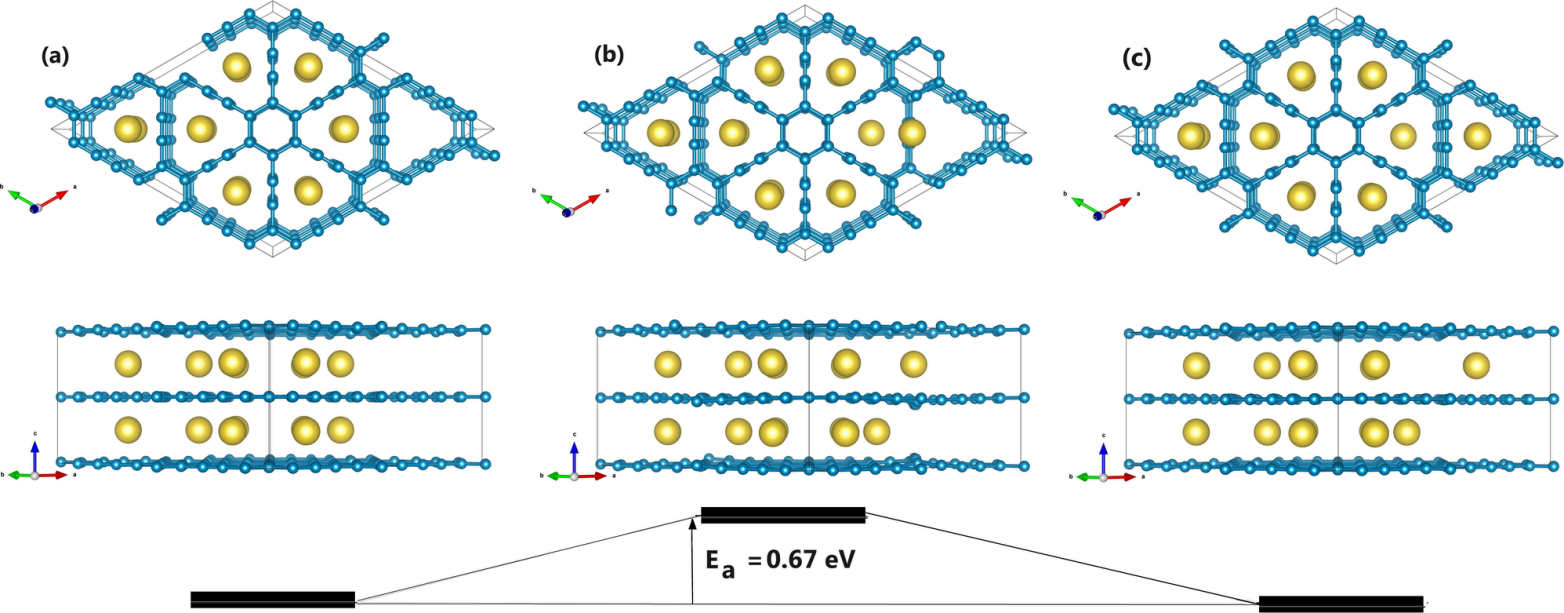
Top and lateral view of the diffusion process of one Na atom in the direction parallel to the carbon plane: (a) reactant, (b) transition state, and (c) product. The Na atom migrating toward the unoccupied pore is located in the top layer. In the transition state the Na atom is lying in correspondence of the midpoint of the acetylenic linkage separating the adjacent pores. [gold, Na; light blue, C atoms].

A final consideration concerns the electrical conductivity of the system of interest, and accordingly, the Projected Density of States (pDOS) has been calculated and reported in Figure 5. In particular, the DFT-1/2 approach, previously tested on the diamond crystal (see S.I.), provides a bandgap for pristine multilayer graphyne in the AB stacking of 0.385 eV (see Figure 5b), which is in good agreement with the experimental value of 0.48 eV.^21^ However, when we move from the AB to the AA stacking, the closure of the bandgap is observed (see Figure 5c) which is associated with the metallization of the system. Similarly to the case of Li-GIC, Na intercalation in graphyne induces an enhancement of the electron conductivity. The occurrence of Na 3s orbitals in the conduction region has indeed the effect to push the C 2s contribution toward the Fermi level (see Figure 5d), thus enhancing electrical conductivity in Na-GYIC, further testifying to the suitability of graphyne as material for anode in Na batteries. Importantly, the fingerprint of the enhanced interaction between Na and C atoms can also be observed from the pDOS analysis of such a system, as reported in Figure S9 of S.I.

**Figure 5:**
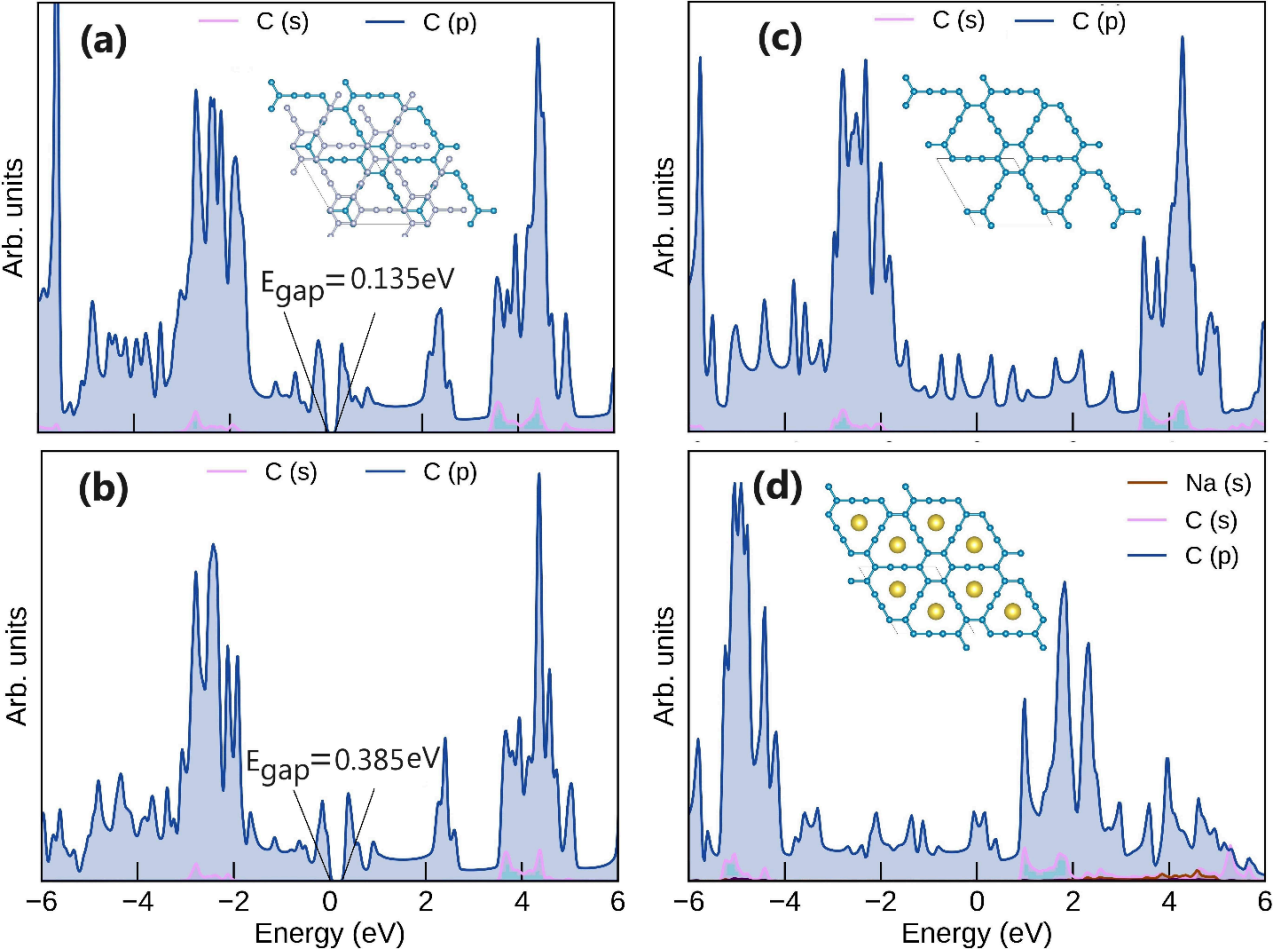
(a) DFT calculated pDOS for AB stacked pristine graphyne; (b) DFT-1/2 calculated pDOS for AB stacked pristine graphyne; (c) DFT calculated pDOS for pristine AA stacked graphyne; (d) DFT calculated pDOS for AαAα stacked Na-GYIC.

In conclusion, we have proposed the validation of the recently synthesized multilayered γ-graphyne as an optimal host for Na atoms intercalation in a similar manner as it occurs for graphite with Li atom counterparts. By means of accurate ab initio electronic structure calculations we have demonstrated that, unlike what is observed in graphite, Na intercalation in multilayer γ-graphyne is thermodynamically stable. This is related to the enhancement of the metal–substrate coupling, which is a consequence of the porous structure of γ-graphyne that allows Na atoms to lie closer to the carbon plane. Indeed, the γ-graphyne pore has an ”ad hoc” size to accommodate a single Na atom leading to an optimal binary intercalation compound of NaC_6_ stoichiometry, which is matching that (LiC_6_) theoretically obtained for the maximum gravimetric storage capacity (372 mAh·g^–1^) of Li in graphite. Moreover, it is predicted that upon metal intercalation the Na-GYIC undergoes a limited deformation of the crystal structure with an interlayer expansion of about 10%, which is comparable with that observed for Li in graphite. This is a key point for its potential use as an electrode since a large volume change during the charge/discharge cycles can lead to strain and fracture of the material. An estimation of the metal diffusion, which occurs in the direction parallel to the carbon plane, has also evidenced that the Na mobility in Na-GYIC is slightly larger but comparable to that for Li in Li-GIC. In a possible application as an anode in metal ion batteries, this represents an important feature, and the slightly poorer metal mobility performance of Na-GYIC within the crystal could be, in principle, compensated by a lower desolvation energy for the Na ion (with respect to Li), related to its smaller solvation radius, and required to transfer the naked ion across the liquid/solid interfaces. Finally, we have demonstrated that a bandgap closure occurs for the multilayered carbon host in the AA stacking as well as an enhancement of the electrical conductivity upon Na atoms intercalation.

Advances in γ-graphyne synthesis^30^ are expected to increase in the upcoming years and we believe that the present study, properly supported by an experimental validation, could pave the way to the development of more efficient Na-ion batteries, which are paramount for meeting the increasing need of high-performance and cheaper energy storage devices.

## Theoretical Methods

A detailed explanation of the electronic structure calculations for the finite and periodic models of the systems considered in this study is provided in the Supporting Information.

## Supporting Information Available

Additional figures and one table showing complementary results as well as more details on the performed calculations. (PDF)
