# Supplementary material for: Sodium into γ-Graphyne Multilayers: An Intercalation Compound for Anodes in Metal-Ion Batteries
Source: ACS Mater Lett. 2024 Sep 12;6(10):4682–9. doi: 10.1021/acsmaterialslett.4c01119 (PMC11463692; doi:10.1021/acsmaterialslett.4c01119)
Supplement: Supplementary file 1 — tz4c01119_si_001.pdf [file tz4c01119_si_001.pdf]

Supporting Information:

Sodium into γ-Graphyne Multilayers: an Intercalation Compound for Anodes in Metal-Ion Batteries

Massimiliano Bartolomei*,† and Giacomo Giorgi*,‡,¶,§

†*Instituto de Física Fundamental, Consejo Superior de Investigaciones Científicas (IFF-CSIC), Serrano 123, 28006, Madrid, Spain*

‡*Department of Civil and Environmental Engineering (DICA), Università degli Studi di Perugia, Via G. Duranti 93, 06125, Perugia, Italy*

¶*CNR-SCITEC, I-06123, Perugia, Italy*

§*CIRIAF - Interuniversity Research Centre, University of Perugia, Perugia, Italy*

E-mail: maxbart@iff.csic.es; giacomo.giorgi@unipg.it

Electronic structure calculations for the finite models

Electronic structure calculations of the interaction energies between the alkali ion and graphene /graphyne finite prototypes have been performed at the MP2 and DFT levels of theory by using the Molpro2012.1[1–3] and Gaussian09[4] codes, respectively. In the MP2 computations the aug-cc-pVTZ[5] and def2-AQZVPP[6] basis sets have been used for the planar prototype and alkali ion, respectively. In the DFT calculations the Perdew-Becke-Ernzerhof (PBE)[7] functional has been employed together with the D3(BJ) dispersion correction contribution

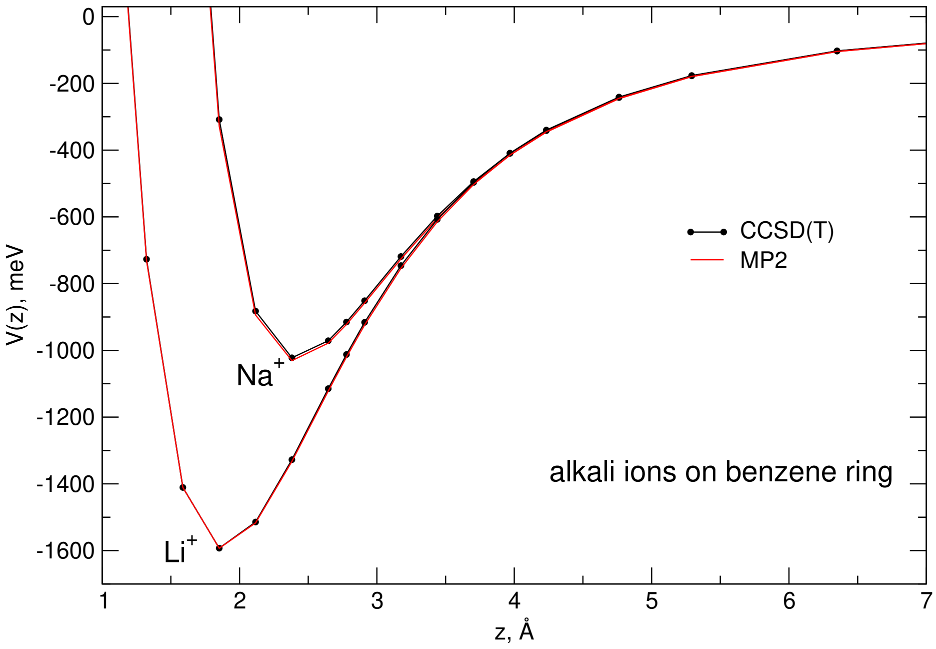

FIG. S1. Benchmark interaction energies obtained at the CCSD(T) and MP2 level of theory together with the aug-cc-pVTZ and def2-ATZVPP basis sets (for the hydrocarbon and alkali atom, respectively) for Li^+ and Na^+ ions perpendicularly approaching the geometric center of benzene.

of Grimme,[8] which includes the damped dispersion scheme of Becke-Johnson (BJ),[9] and combined with the 6-311+G[10] basis set.

All interaction energies have been corrected for the basis set superposition error (BSSE) by the counterpoise method of Boys and Bernardi.[11] The obtained interaction energy profiles refer to a rigid monomers' approximation, that is the configuration of the graphene/graphyne finite prototype is not allowed to relax during the calculations.

The estimations of the alkali ion-carbon support interaction energy obtained at the PBE-D3(BJ) level of theory are reported in Figure S2, where they are compared with the benchmark results from Figure 2 of the main maunscript. It can be appreciated that the DFT "hollow"-"bridge" energy gaps are estimated to be around 0.20 and 0.54 eV for Li^+ and

Na^+, respectively, and therefore very close to the related benchmark values of 0.24 and 0.55 eV. The same DFT approach was found adequate to describe both K^+-coronene[12] and coronene-coronene[13] interactions, the latter being a prototypical system dominated by π-π interactions, which are those responsible for the stacking of graphene layers in graphite. Therefore, we believe that the PBE-D3(BJ) approach is appropriate to be used in DFT periodic calculations aimed to to assess the energy and structure of 3D layered carbon materials as host for alkali metals through intercalation. Indeed, the same approach has been recently used[14] to describe the features of different alkali metal-GICs.

Electronic structure calculations for the periodic models

In the periodic calculations the PBE[7] functional as implemented in the VASP code[15–18] has been employed. The projector-augmented wave (PAW) potentials,[19] with an energy cutoff of 700 eV, have been adopted. In analogy with the finite prototype estimations, the D3(BJ) dispersion correction contribution has been included in all the calculations. Geometries were considered converged when forces were lower than 0.01 eV/Å. As for the case of graphene (monolayer) and graphite (stacked multilayer), a massive number of k-points (as function of the size of the system) were included to sample the Brilloiun Zone. A bilayered unit cell has been considered to describe the binary graphite/graphyne intercalation compound (GIC/GIYC) crystal structures: for the former compound the optimized lateral parameters are a=b=4.31 Å,[20] while for the latter compound are a=b=6.87Å. The two related structures are reported in Fig. S4.

For the subsequent analysis of the alkali diffusion within the layered materials, we have considered larger unit cells, that is a 2×2 bilayer for GYIC (shown in Fig. 4 of the main text) and a 2×4 bilayer for GIC (shown in Fig. S6).[21]

The electronic energy values needed to estimate the formation energy (see Eq. 1 in the main text) have been obtained from crystal structure optimizations, which led to stationary

point configurations. Energy differences between stationary point configurations have been also used to estimate the diffusion barriers for the alkali ion migration. In these cases we have checked that, by exploiting vibrational frequency calculations in a reduced finite model, the intermediate configurations in Fig. 4 of the main text and Fig. S6 correctly correspond to transition states. The analysis of the atomic charges was performed by means of the Bader code.[22–25]

Interaction energies between one isolated Na atom and a graphyne single layer are reported in Figure S3, where it can be appreciated that the estimated well depth and equilibrium distance are in good agreement with those reported in the right panel of Figure S2 and related to the finite graphyne prototype.

In order to assess the change of the stacking configuration upon Na intercalation we have optimized and analyzed the GYIC in the following configurations:

1. AαAα (see Fig. S4(b))

2. AαBα (see inset in Figure 5(a) in the main text)

3. AαBα (alternative fashion, see Fig. S4(c) and inset in Fig. S8(a))

While the first configuration (**1.**) characterizes all the Na intercalation compounds discussed throughout the paper, the second one (**2.**), obtained by gliding the top "A" layer in order to form a Bernal-like AB stacking, did not lead to any stable intercalated structure. The third configuration (**3.**), which represents an alternative AB stacking, leads to an intercalation compound that is less stable (compared to **1.**) by 0.40 eV, suggesting therefore a transition from the AB to AA stacking upon Na intercalation.

The dependence of the formation energy (see Eq. 1 in the main manuscript) on the density of the Na intercalated into the graphyne multi-layer has been also determined and reported in Fig. S5: an increasing number of Na atoms has been considered, from the (n_{top}, n_{bottom}) = (1,2) to (n_{top}, n_{bottom}) = (4,4) configurations (n_{top} and n_{bottom} are the number of

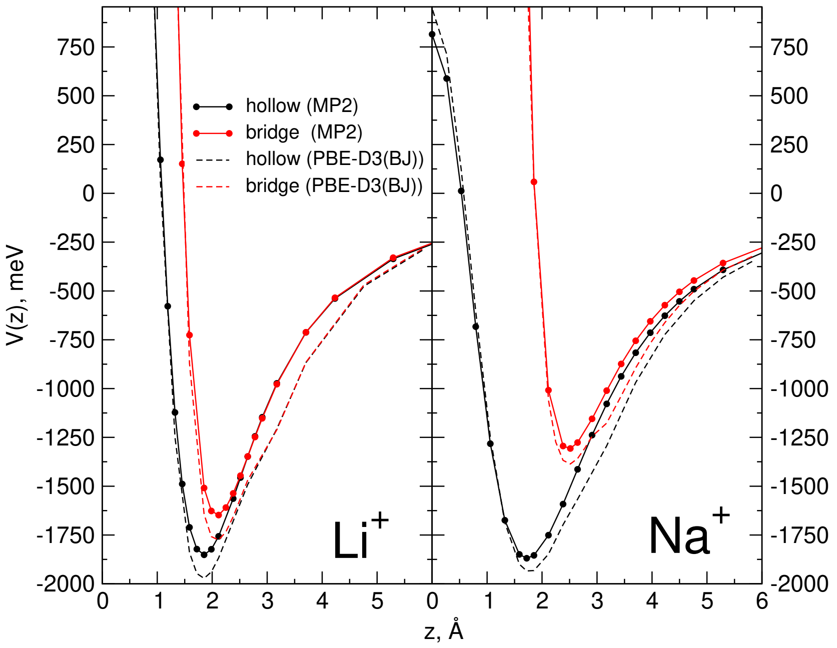

FIG. S2. As in Fig. 2 of the main manuscript but with the inclusion of PBE-D3(BJ) results reported as dashed lines.

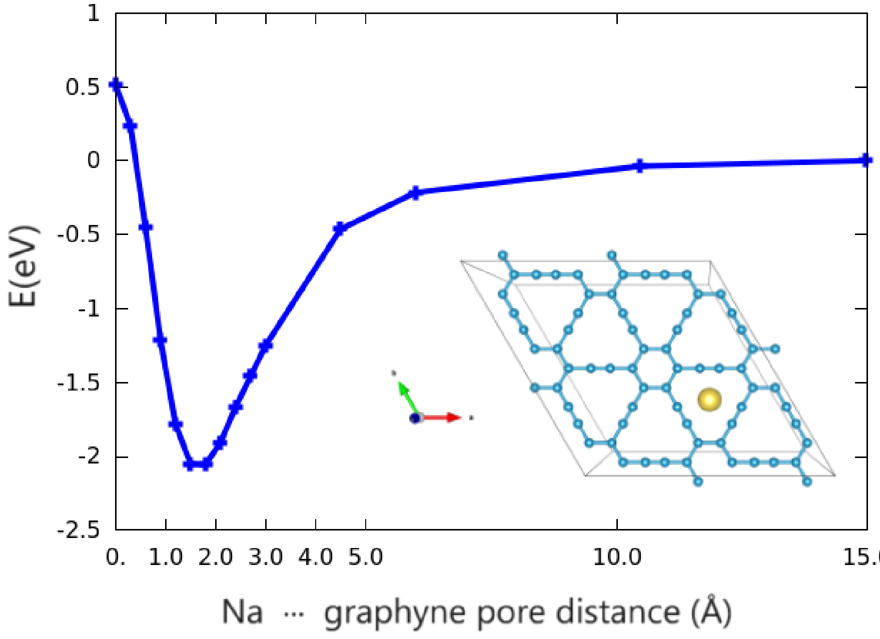

FIG. S3. Periodic DFT interaction energy (eV) of one isolated Na atom perpendicularly approaching the pore geometric center of a graphyne single layer.

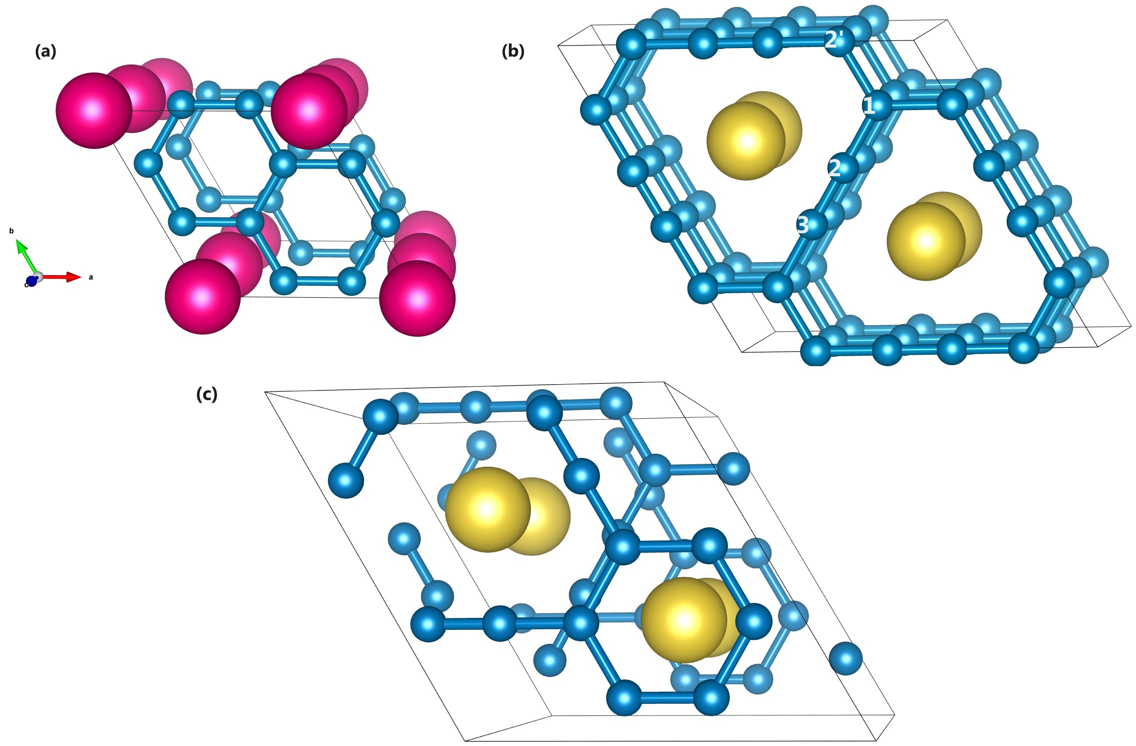

FIG S4. GIC (a) and GYIC (b) structures in the AαAα stacking. (c) GYIC structure in the alternative AαBα stacking. The latter structure is less stable than (b) by $\sim$ 0.40 eV [Purple, alkali metal atom; Gold, Na atom; Light blue, C atoms]. Numbering in (b) is intended as a tool to facilitate the interpretation of the results in Table S1.

Na atoms adsorbed on the top and bottom layer, respectively, of the bulk graphyne unit cell). One can easily notice that a minimum in the formation energy appears for the (2,2) configuration, *i.e.*, that with each graphyne pore hosting a single Na atom. For larger Na densities, obtained by occupying with additional Na atoms the phenyl ring sites and the midpoint of the acetylenic linkages, not only the formation energy becomes less and less favourable, but it is also accompanied by an appreciable increase of the interlayer distance of about 22% and 42% for the (3,3) and (4,4) cases, respectively, compared to the pristine host material. Such an important crystal deformation is indeed not desirable since could lead to more important thermal and volume effects (*swelling*) during the charge/discharge cycles. Therefore, the reported theoretical storage capacity of 372 mAh$\cdot$g^{-1}, corresponding to the above mentioned (2,2) configuration and to the NaC_6 stoichiometry, should be considered as an optimal one.

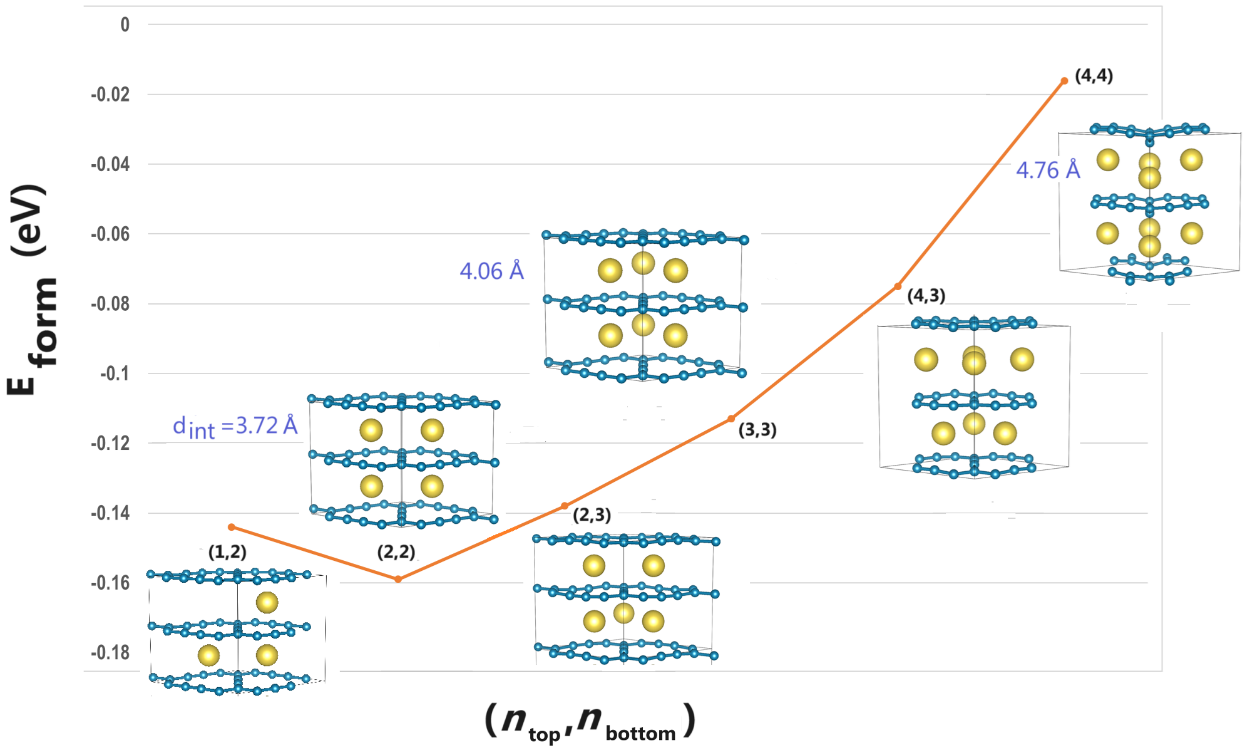

Fig. S5: Formation energy (E_{form}) of the Na-GYIC as a function of n_{top}, n_{bottom}, *i.e.*, the number of Na atoms adsorbed on the top and bottom layer, respectively, of the bulk graphyne unit cell. The interlayer distance (d_{int}) is also reported for even values of both n_{top} and n_{bottom}.

TABLE S1: C-C bond lengths in both GIC and GYIC (compared with the pristine cases) along with their Bader charge analysis. C atom numbering in the case of γ-graphyne systems is same as that of Fig. S4(b). For GICs the C atoms charges are intended to range between the two reported values.

compound	d_{C-C} (Å)	Bader charge
graphite (ABAB)	1.423	C: -0.05 ÷ +0.05/atom (total 0.0)
Li-GIC (AαAα)	1.436	Li: +0.861<br>C: -0.09 ÷ -0.19/atom (total -0.861)
Na-GIC (AαAα)	1.438	Na: +0.864<br>C: -0.13 ÷ -0.17/atom (total -0.864)
γ-graphyne (ABAB)	C_1-C_2=1.403<br>C_1-$C_{2'}$=1.423<br>C_2-C_3=1.221	C_1: +0.12; C_2: -0.31<br>$C_{2'}$: +0.09; C_3: +0.06
Na-GYIC (AαAα)	C_1-C_2=1.388<br>C_1-$C_{2'}$=1.445<br>C_2-C_3=1.255	Na: +0.873<br>C_1: +0.12; C_2: -0.34<br>$C_{2'}$: +0.09; C_3: -0.45 (total -0.873)

Electronic feature calculations for the periodic models

As for the electronic properties of graphyne, we initially calculated the bandstructure and density of states at the PAW/PBE level; however, since it is well known that a pure DFT approach quantitatively underestimates the experimental material gap, we here exploited the DFT$-1/2$,[26] a semi-empirical method recently developed by Teles *et al.* and based on the Slater[27,28] transition state technique. Such a method, specifically developed for the correction of the self-interaction error in local and semi-local exchange-correlation density functionals for extended systems,[26] is characterized by a precision similar to that of methods based on the Green function and on hybrid functionals[29,30] at a massively reduced computational cost. In particular, by defining an atomic self-energy potential able to compensate the electron-

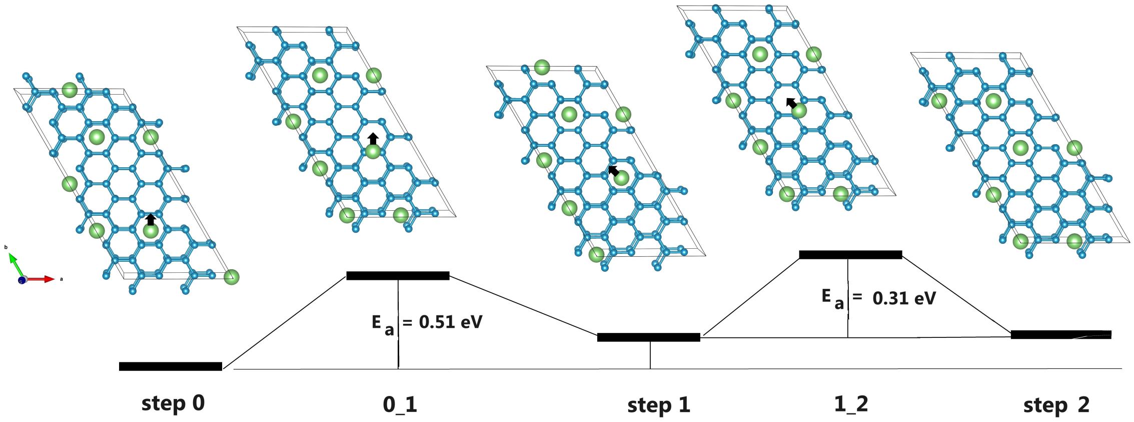

FIG S6. Top view of the alkali diffusion mechanism in the 7/7 Li-GIC bilayer. For the sake of comprehension, the bottom layer of Li atoms is not reported. The black arrow indicates the diffusion path [Green, Li; Light blue, C atoms]

.

hole self-interaction energy (SIE) the method aims to correct the inherent SIE of DFT. The procedure consists of calculating the self-energy which is added to the local part of the PAW pseudopotentials. When extended to the study of crystals, the self-energy potential is cut to avoid its extension to neighboring atoms. Such truncation is performed exploiting a step function with a parameter which is variationally calculated by maximizing the bandgap. The convergence test for both C diamond (to validate the setup) and C graphyne are reported in Figure S7. In particular, it is shown that the DFT-1/2 approach is capable to predict for the bandgap of the diamond crystal a value of 4.99 eV, in very good agreement with previous published result at the same level of theory (5.0 eV)[31] and not far from the experimental value.[32] In addition to the pDOS results reported in Fig. 5 of the main manuscript, those corresponding to an alternative AB stacking are shown in Fig. S8.

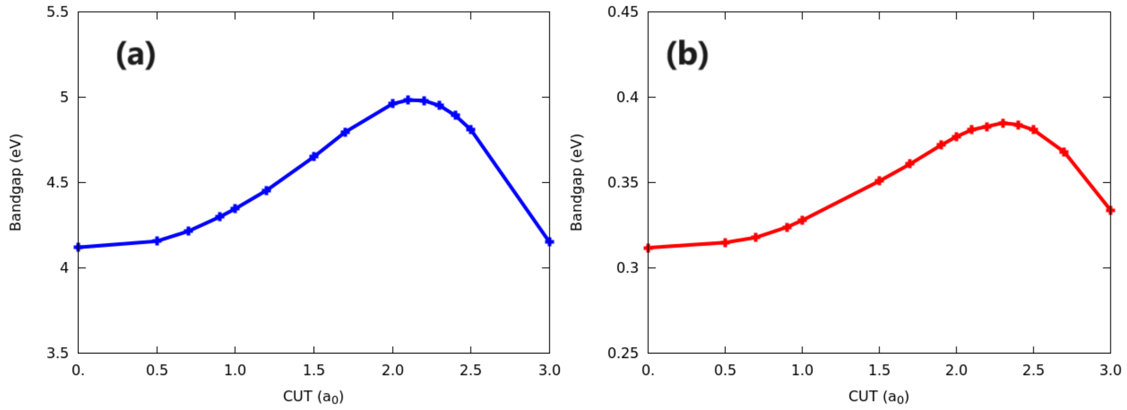

FIG. S7. CUT determination for the DFT-1/2 corrections after 0.25 electron removal from the C atom 2p orbitals of (a) C bulk diamond, (b) graphyne bilayer structure (AB stacking) .

References

(1) Werner, H.-J.; Knowles, P. J.; Knizia, G.; Manby, F. R.; Schütz, M. Molpro: a general-purpose quantum chemistry program package. *Wiley Interdiscip. Rev. Comput. Mol. Sci.* **2012**, *2*, 242–253.

(2) Werner, H.-J.; Knowles, P. J.; Manby, F. R.; Black, J. A.; Doll, K.; Heßelmann, A.; Kats, D.; Köhn, A.; Korona, T.; Kreplin, D. A., et al. The Molpro quantum chemistry package. *J. Chem. Phys.* **2020**, *152*, 144107–24.

(3) MOLPRO, version 2012.1 , a package of ab initio programs, H.-J. Werner, P. J. Knowles, G. Knizia, F. R. Manby, M. Schütz, and others, see https://www.molpro.net (accessed 2024-08-23).

(4) M. J. Frisch, G. W. Trucks, H. B. Schlegel, G. E. Scuseria, M. A. Robb, J. R. Cheeseman,G. Scalmani, V. Barone, B. Mennucci, G. A. Petersson, et al., Gaussian 09 Revision E.01, gaussian Inc. Wallingford CT 2009, see https://www.gaussian.com (accessed 2024-08-23).

(5) Kendall, R. A.; Dunning Jr, T. H.; Harrison, R. J. Electron affinities of the first-row

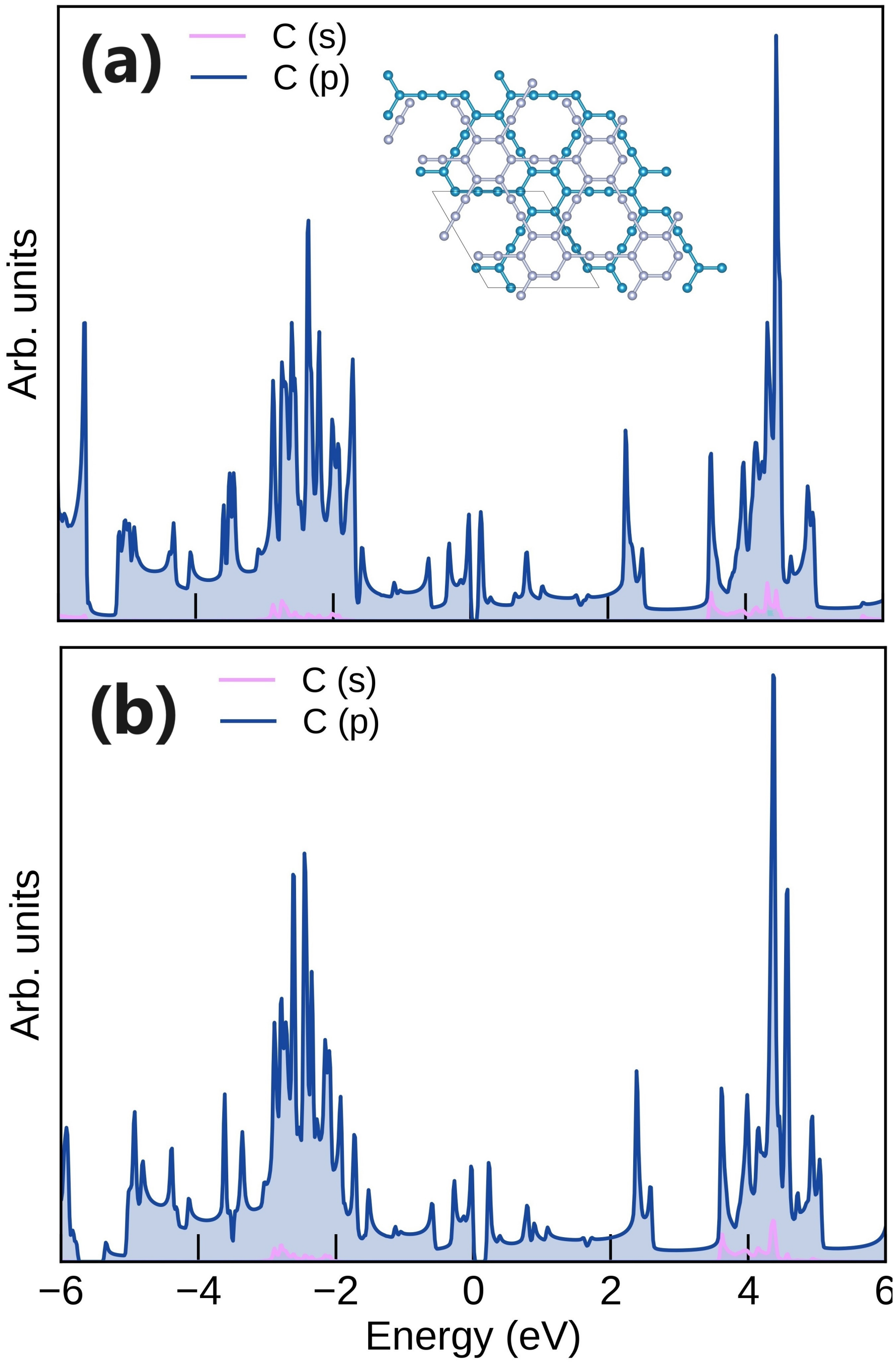

FIG. S8. (a) DFT calculated pDOS for the pristine graphyne bilayer in an alternative AB stacking; (b) Same as above but with DFT-1/2 calculated pDOS

.

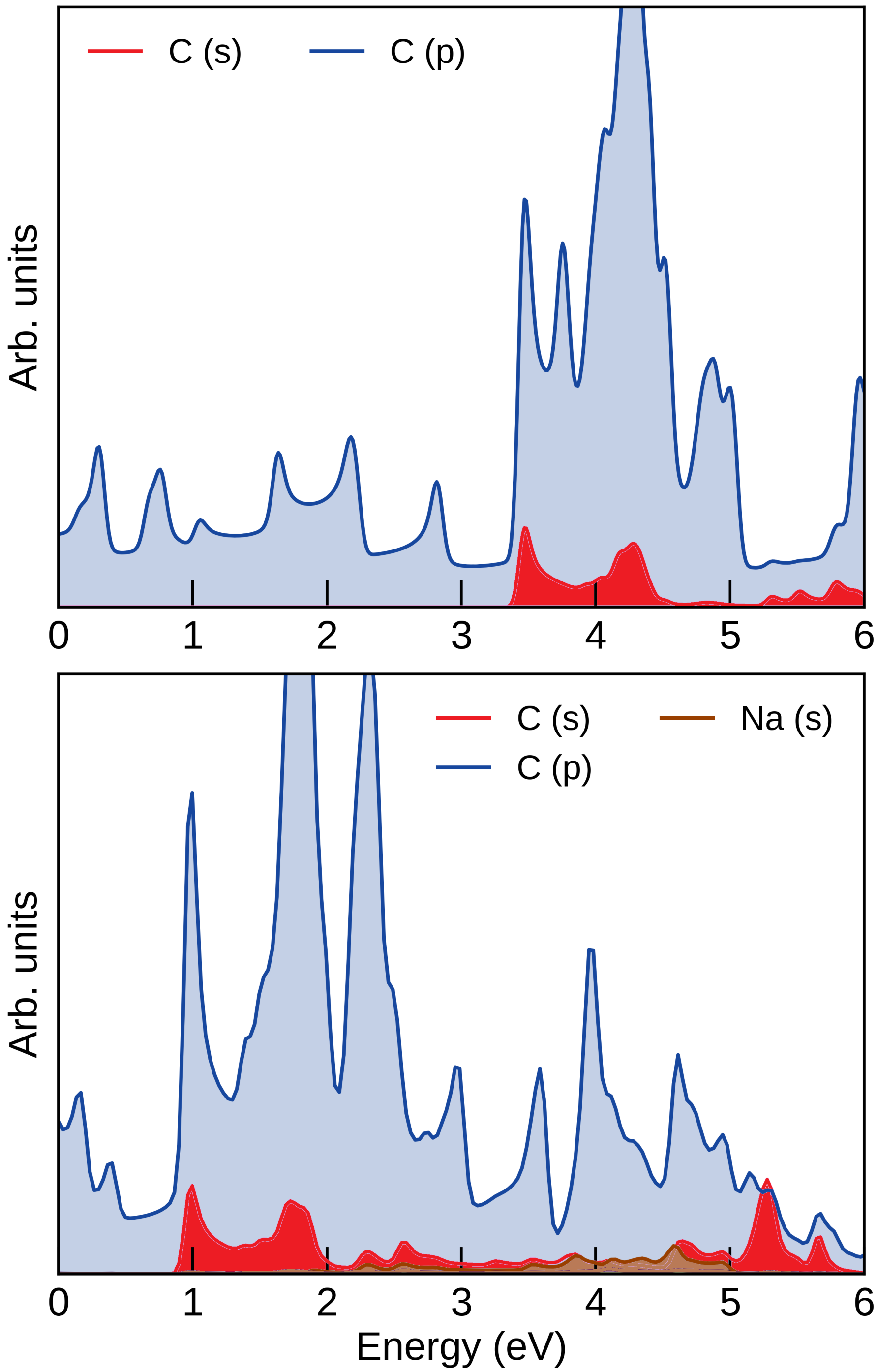

FIG. S9. Zooming on the conduction region for (a) the DFT calculated pDOS for pristine AA stacked graphyne; (b) DFT calculated pDOS for AαAα stacked Na-GYIC.

.

atoms revisited. Systematic basis sets and wave functions. *J. Chem. Phys.* **1992**, *96*, 6796–6806.

(6) Weigend, F.; Ahlrichs, R. Balanced basis sets of split valence, triple zeta valence and quadruple zeta valence quality for H to Rn: Design and assessment of accuracy. *Phys. Chem. Chem. Phys.* **2005**, *7*, 6796–6806.

(7) Perdew, J. P.; Burke, K.; Ernzerhof, M. Generalized gradient approximation made simple. *Phys. Rev. Lett.* **1996**, *77*, 3865–3868.

(8) Grimme, S.; Antony, J.; Ehrlich, S.; Krieg, H. A consistent and accurate ab initio parametrization of density functional dispersion correction (DFT-D) for the 94 elements H-Pu. *J. Chem. Phys.* **2010**, *132*, 154104–19.

(9) Johnson, E. R.; Becke, A. D. A post-Hartree-Fock model of intermolecular interactions: Inclusion of higher-order corrections. *J. Chem. Phys.* **2006**, *124*, 174104–9.

(10) Binkley, J. S.; Pople, J. A.; Hehre, W. J. Self-consistent molecular orbital methods. 21. Small split-valence basis sets for first-row elements. *J. Am. Chem. Soc.* **1980**, *102*, 939–947.

(11) Boys, S. F.; Bernardi, F. The calculation of small molecular interactions by the differences of separate total energies. Some procedures with reduced errors. *Mol. Phys.* **1970**, *19*, 553–566.

(12) Bartolomei, M.; Pirani, F.; Marques, J. Aggregation enhancement of coronene molecules by seeding with alkali-metal ions. *Phys. Chem. Chem. Phys.* **2019**, *21*, 16005–16016.

(13) Bartolomei, M.; Pirani, F.; Marques, J. Modeling coronene nanostructures: analytical potential, stable configurations and ab initio energies. *J. Phys. Chem. C* **2017**, *121*, 14330–14338.

(14) Lenchuk, O.; Adelhelm, P.; Mollenhauer, D. New insights into the origin of unstable sodium graphite intercalation compounds. *Phys. Chem. Chem. Phys* **2019**, *21*, 19378–19390.

(15) Kresse, G.; Hafner, J. Ab initio molecular dynamics for open-shell transition metals. *Phys. Rev. B* **1993**, *48*, 13115–13118.

(16) Kresse, G.; Hafner, J. Ab initio molecular-dynamics simulation of the liquid-metal–amorphous-semiconductor transition in germanium. *Phys. Rev. B* **1994**, *49*, 14251–14269.

(17) Kresse, G.; Furthmüller, J. Efficiency of ab-initio total energy calculations for metals and semiconductors using a plane-wave basis set. *Comput. Mat. Sci.* **1996**, *6*, 15–50.

(18) Kresse, G.; Furthmüller, J. Efficient iterative schemes for ab initio total-energy calculations using a plane-wave basis set. *Phys. Rev. B* **1996**, *54*, 11169–11186.

(19) Blöchl, P. E. Projector augmented-wave method. *Phys. Rev. B* **1994**, *50*, 17953–17979.

(20) Imai, Y.; Watanabe, A. Energetic evaluation of possible stacking structures of Li-intercalation in graphite using a first-principle pseudopotential calculation. *J. Alloys Compd.* **2007**, *439*, 258–267.

(21) Persson, K.; Hinuma, Y.; Meng, Y. S.; Van der Ven, A.; Ceder, G. Thermodynamic and kinetic properties of the Li-graphite system from first-principles calculations. *Phys. Rev. B* **2010**, *82*, 125416–9.

(22) Tang, W.; Sanville, E.; Henkelman, G. A grid-based Bader analysis algorithm without lattice bias. *J. Phys. Condens. Matter* **2009**, *21*, 084204–7.

(23) Sanville, E.; Kenny, S. D.; Smith, R.; Henkelman, G. Improved grid-based algorithm for Bader charge allocation. *J. Comput. Chem.* **2007**, *28*, 899–908.

(24) Henkelman, G.; Arnaldsson, A.; Jónsson, H. A fast and robust algorithm for Bader decomposition of charge density. *Comput. Mater. Sci.* **2006**, *36*, 354–360.

(25) Yu, M.; Trinkle, D. R. Accurate and efficient algorithm for Bader charge integration. *J. Chem. Phys.* **2011**, *134*, 064111–8.

(26) Ferreira, L. G.; Marques, M.; Teles, L. K. Approximation to density functional theory for the calculation of band gaps of semiconductors. *Phys. Rev. B* **2008**, *78*, 125116–9.

(27) Ferreira, L. G.; Marques, M.; Teles, L. K. Slater half-occupation technique revisited: the LDA-1/2 and GGA-1/2 approaches for atomic ionization energies and band gaps in semiconductors. *AIP Adv.* **2011**, *1*, 032119–11.

(28) Slater, J. C.; Johnson, K. H. Self-consistent-field X α cluster method for polyatomic molecules and solids. *Phys. Rev. B* **1972**, *5*, 844–853.

(29) Krukau, A. V.; Vydrov, O. A.; Izmaylov, A. F.; Scuseria, G. E. Influence of the exchange screening parameter on the performance of screened hybrid functionals. *J. Chem. Phys.* **2006**, *125*, 224106–5.

(30) Paier, J.; Marsman, M.; Hummer, K.; Kresse, G.; Gerber, I. C.; Ángyán, J. G. Screened hybrid density functionals applied to solids. *J. Chem. Phys.* **2006**, *124*, 154709–33.

(31) Lucatto, B.; Assali, L. V.; Pela, R. R.; Marques, M.; Teles, L. K. General procedure for the calculation of accurate defect excitation energies from DFT-1/2 band structures: the case of the NV- center in diamond. *Phys. Rev. B* **2017**, *96*, 075145–9.

(32) Madelung, O.; Schulz, M.; Weiss, H. Intrinsic properties of group IV elements and III-V, II-VI and I-VII compounds. *Landolt-Bornstei, New Series, Group III* **1987**,
